# Acquired thrombotic thrombocytopenic purpura after AstraZeneca vaccine: A case report

**DOI:** 10.22088/cjim.13.0.299

**Published:** 2022

**Authors:** Fatemeh Yaghoubi, Davood Dalil

**Affiliations:** 1 Nephrology Research Center, Shariati Hospital, Tehran University of Medical Sciences, Tehran, Iran; 2 Medical Student Research Committee, Faculty of Medicine, Shahed University, Tehran, Iran

**Keywords:** COVID-19, Vaccines, Thrombocytopenia, TTP, AstraZeneca

## Abstract

**Background:**

Rare cases of acquired thrombotic thrombocytopenic purpura (aTTP) have been reported since the administration of the COVID-19 vaccination. Based on our information, the present study provides the first case report of aTTP developed after the COVID-19 vaccination in Iran.

**Case presentation:**

A 22‐year‐old Iranian woman presented with symptoms of ataxia, dysphasia, paresthesia, and acute numbness of her left upper limb four weeks after the AstraZeneca COVID-19 vaccination. Laboratory data suggested hemolytic anemia and thrombocytopenia. Also, schistocytes were noted on her peripheral blood smear. Acquired thrombotic thrombocytopenic purpura (aTTP) was diagnosed in accordance with clinical manifestations along with initial blood test results and was confirmed later through findings of ADAMTS-13 low level activity and the ADAMTS-13 positive inhibitor. She underwent 22 sessions of plasma exchange, receiving corticosteroid and rituximab. Finally, the treatment was successful.

**Conclusion:**

Despite the presence of rare complications such as aTTP, vaccination is one of the best ways to prevent COVID-19 disease. The present case report describes the potential, but unproven, role of the AstraZeneca COVID-19 vaccine in aTTP pathogenesis. Vaccine-associated aTTP can be successfully treated with plasma exchange, corticosteroids, and rituximab.

Vaccination is one of the effective efforts of global scientific community to end the novel 2019 coronavirus disease (COVID-19) pandemic. To date, the World Health Organization (WHO) has approved eight vaccines for emergency use: Pfizer/BioNTech, Moderna, AstraZeneca, Johnson and Johnson, Covishield, Sinovac, Sinopharm, and Bharat Biotech (1). Among these, the AstraZeneca vaccine, undergoing 47 clinical trials in 23 countries, has been recognized as one of the most effective, efficient, and safe vaccines, which has been approved and used by 124 countries so far (2). Since the administration of the vaccine, an immune-mediated thrombotic thrombocytopenia syndrome has been reported in several individuals worldwide (3-6). Acquired thrombotic thrombocytopenic purpura (aTTP) is a rare hematologic disorder characterized by thrombotic microangiopathies causing multi-organ ischemia, including the cerebrum mainly and to lesser extent renal and cardia. This report documented the case of aTTP diagnosed after administration of AZD1222 Vaxzevria (AstraZeneca) vaccine. 

## Case Presentation

A 22-year-old Iranian woman admitted to the emergency ward of Shariati Hospital, Tehran, Iran, with symptoms of ataxia, dysphasia, paresthesia, and acute numbness of her left upper extremity. She had not history of any serious and remarkable diseases. Four weeks before her current hospital admission, she received the first dose of AZD1222 Vaxzevria (AstraZeneca) COVID-19 vaccine, and her symptoms began three weeks later.

On examination, the patient was alert and oriented. Her temperature, O2 saturation, and respiratory rate were normal. But her systolic blood pressure was 180 mmHg. Initial blood tests revealed hemolytic anemia (hemoglobin 10.8 g/dL, normal range 120-160 g/dL, reticulocyte index 3.2% >2.5%) and a low platelet count (45×109/L, normal range 150–400×109/L). The erythrocyte sedimentation rate (ESR) was high (77 mm/hr, normal range 0-30 mm/hr) and D-dimer was mildly elevated (424 ng/mL). Also, the serum fibrinogen was within the normal range (235 mg/dL). Her peripheral blood smear (PBS) test showed 5-6 percent schistocytes. Thus, the PBS confirmed thrombocytopenia with microangiopathic hemolytic anemia characterized by schistocytes. Based on these early findings, aTTP with cerebral thrombosis was the initial diagnosis.

Further tests demonstrated an increase in the following parameters: total bilirubin (2.2 mg/dL, normal range 0.3-1.2 mg/dL; direct bilirubin 1.8 mg/dL), lactate dehydrogenase (LDH) (809 U/L, normal range 81–234 U/L), uric acid (9.5 mg/dL, normal range 2.7-7.3 mg/dL for adult female), and serum creatinine (6.8 mg/dL, normal range 0.59-1.04 mg/dL for adult female). Fluorescent antinuclear antibody (FANA) and lupus anticoagulant were both negative. Chest x-ray, cardiac echo, and renal sonography were regular. The nasopharyngeal swab RT-PCR testing for COVID-19 was negative.

**Fig 1:**
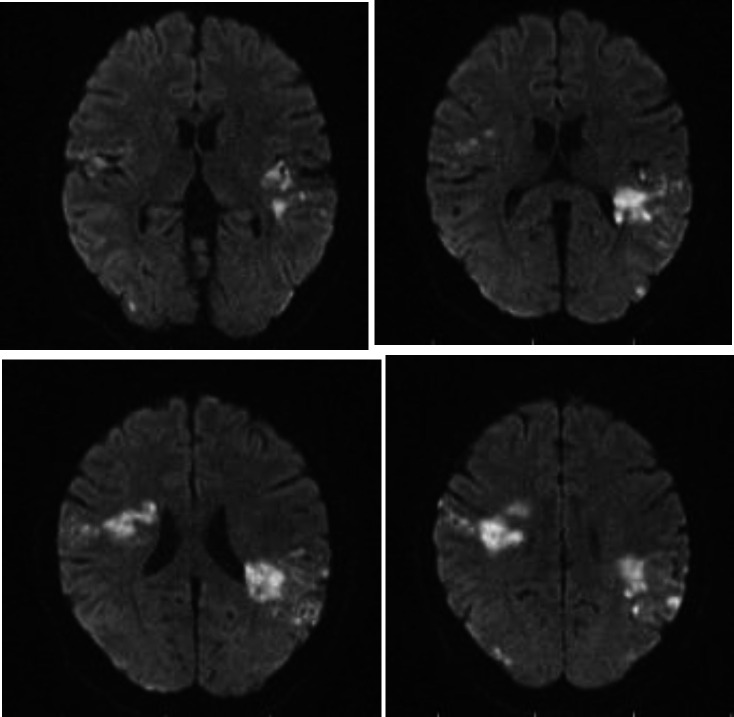
Brain MRI without gadolinium; Multiple acute infarction areas in left temporoparietal, right temporofrontal and right parietal cortex are seen

**Fig 2:**
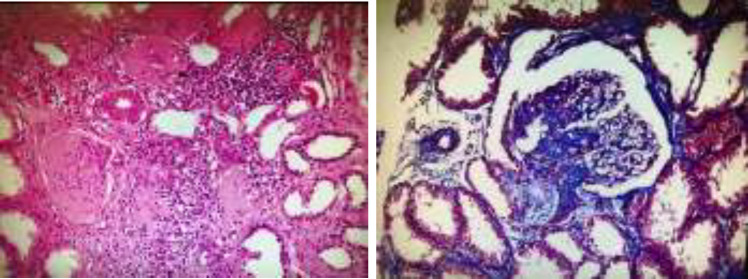
Kidney needle biopsy, (A) Hematoxylin and Eosin stain, (B) Masson's trichrome stain; MPGN like glomerular changes with obliterated small arteries consistent with thrombotic microangiopathy

Suspected of having a stroke based on the symptoms of her neurological disorder, a brain MRI was requested. Brain MRI (without gadolinium) reported multiple acute infarction areas in the left temporoparietal, right temporofrontal, and right parietal cortex (fig 1). According to creatinine rise with thrombocytopenia and brain infarction along with hematuria and proteinuria, a kidney needle biopsy (KNB) was asked. The KNB reported glomerular changes like membranoproliferative glomerulonephritis (MPGN) with obliterated small arteries consistent with thrombotic microangiopathy (fig 2).

The diagnosis of aTTP was later confirmed by ADAMTS13 activity assay and inhibitor profile. ADAMTS13 activity was 0.4% (<10 %) and ADAMTS13 inhibitor was positive (6.5), which is highly indicative of thrombotic thrombocytopenic purpura. The platelet factor 4 (PF4)-heparin antibodies, which are found in heparin-induced thrombocytopenia, were negative.

**Treatment and Outcome** **: **The patient was admitted to the intensive care unit (ICU) of Shariati Hospital and treatment was started. During hospitalization, she underwent 22 times plasmapheresis with fresh frozen plasma along with 3 pulses of intravenous methylprednisolone 500 mg/kg and rituximab in the first week and then prednisolone 60 mg daily. The procedures were well tolerated and increased the platelet count to 365×109/L. The platelet counts also remained within the normal range after cessation of plasmapheresis.

Due to the presence of neurological symptoms, the patient was examined by the neurology service once she was admitted to the hospital. After examination, the patient was prescribed aspirin and clopidogrel and was not required to receive a tissue plasminogen activator (TPA). Also, to manage the creatinine rise with proteinuria, three times of hemodialysis were performed for the patient which led to creatinine decline to 2 mg/dL. Four weeks after hospitalization, the patient was discharged on reduction dosage of oral prednisolone 1 mg/kg and aspirin for a month later.

## Discussion

aTTP is a rare blood disorder caused by a severe reduction in ADAMTS13 (von Willebrand factor-cleaving protease) activity due to inhibitory antibodies. The aTTP annual incidence is 3 to 10 cases per million adults (7). The disease is determined by microangiopathic hemolytic anemia (characterized by schistocytes on the PBS), thrombocytopenia, and thrombosis. In the progressive and aggressive stages of aTTP, mostly the central nervous system (CNS) suffers from the ischemic condition, and the patient may experience any neurological dificits including headache, ataxia, dysphasia, paresthesia, strokes, transient ischemic attack (TIA), coma, seizure, or confusion (8). 

Vaccination, generally, plays a significant role in preventive medicine. However, very rare complications have been reported after receiving the vaccine, especially against viral infection, including autoimmune disorders such as TTP. Previously, several case reports documented TTP after administering the influenza vaccine^9^, pneumococcal vaccine (10), after H1N1 vaccination^11^, and after rabies vaccination (12). In the current pandemic, it is well proven that the COVID-19 vaccination considerably reduces the mortality and morbidity of the disease. Nevertheless, a rare thrombotic thrombocytopenia disorder has been reported worldwide since the COVID-19 vaccination. Since April 2021, a newly vaccine-induced immune thrombotic thrombocytopenia (VITT) syndrome has been reported in several individuals after the COVID-19 vaccination. Schultz et al. reported five cases of VITT after vaccination with ChAdOx1 nCoV-19 (4). Greinacher et al. also documented 11 cases of VITT in Germany and Austria (5). They demonstrated that VITT imitates heparin-induced thrombocytopenia through PF4-heparin antibodies (4, 5). Al-Ahmad et al. and Lee et al. reported the thrombotic thrombocytopenic purpura after the AstraZeneca COVID‐19 vaccine injection in a 37 year-old man and 50 year-old woman, respectively (3, 6).

In this report, the patient has been referred to the emergency department with focal CNS signs three weeks after vaccination with the AZD1222 Vaxzevria (AstraZeneca). In this case, the risk of connective tissue disorders such as systemic lupus erythematosus (SLE) and antiphospholipid syndrome, which is followed by ischemic manifestations, was eliminated by lack of FANA, and negative lupus anticoagulation. Also, the VITT was ruled out in this patient due to the lack of expected evidence in VITT, such as antibodies to PF4–polyanion complexes, markedly increased level of D-dimer and depleted fibrinogen. The presence of constant features of aTTP, including microangiopathic hemolytic anemia along with thrombocytopenia and hemolysis markers, strongly raised suspicion of aTTP. Subsequently, aTTP disorder was confirmed by drastic reduction in ADAMTS13 activity and positive ADAMTS13 inhibitor titer.

TTP treatment should begin immediately as soon as it is diagnosed. To normalize the platelet count, daily plasmapheresis is the main part of TTP management. The pathogenic autoantibodies causing ADAMTS13 activity decline will be eliminated by plasmapheresis and normal ADAMTS13 will be replaced. Along with plasmapheresis with FFP, corticosteroids, and rituximab were prescribed to inhibit the ADAMTS13 autoantibodies production (13). Sthat along with the few reports worldwide, possibly associates receiving AZD1222 Vaxzevria (AstraZeneca) vaccine to aTTP. The patient had no past medical history of any diseases and no medication. she only received the COVID-19 vaccination about four weeks before hospital administration.

## References

[1] World Health Organization Status of COVID-19 Vaccines within WHO EUL/PQ evaluation process 2021.

[2] Oxford/AstraZeneca: Vaxzevria Covid 19 Vaccine Tracker 2020.

[3] Al‐Ahmad M, Al‐Rasheed M, Shalaby NAB (2021). Acquired thrombotic thrombocytopenic purpura with possible association with AstraZeneca‐Oxford COVID‐19 vaccine. EJHaem.

[4] Schultz NH, Sørvoll IH, Michelsen AE (2021). Thrombosis and thrombocytopenia after ChAdOx1 nCoV-19 vaccination. New Engl J Med.

[5] Greinacher A, Thiele T, Warkentin TE (2021). Thrombotic thrombocytopenia after ChAdOx1 nCov-19 vaccination. New Engl J Med.

[6] Lee HP, Selvaratnam V, Rajasuriar JS (2021). Thrombotic thrombocytopenic purpura after ChAdOx1 nCoV-19 vaccine. BMJ Case Rep.

[7] Reese JA, Muthurajah DS, Kremer Hovinga JA (2013). Children and adults with thrombotic thrombocytopenic purpura associated with severe, acquired Adamts13 deficiency: comparison of incidence, demographic and clinical features. Pediatr Blood Cancer.

[8] Page EE, Kremer Hovinga JA, Terrell DR, Vesely SK, George JN (2017). Thrombotic thrombocytopenic purpura: diagnostic criteria, clinical features, and long-term outcomes from 1995 through 2015. Blood Adv.

[9] Ramakrishnan N, Parker L (1998). Thrombotic thrombocytopenic purpura following influenza vaccination-- a brief case report. Conn Med.

[10] Kojima Y, Ohashi H, Nakamura T (2014). Acute thrombotic thrombocytopenic purpura after pneumococcal vaccination. Blood Coagul Fibrinolysis.

[11] Hermann R, Pfeil A, Busch M (2010). Very severe thrombotic thrombocytopenic purpura (TTP) after H1N1 vaccination. Med Klin.

[12] Kadikoylu G, Yavasoglu I, Bolaman Z (2014). Rabies vaccine‐associated thrombotic thrombocytopenic purpura. Transfus Med.

[13] Joly BS, Coppo P, Veyradier A (2017). Thrombotic thrombocytopenic purpura. Blood.

